# Fabrication of Hybrid Electrodes by Laser-Induced Forward Transfer for the Detection of Cu^2+^ Ions

**DOI:** 10.3390/ma16041744

**Published:** 2023-02-20

**Authors:** Anca Florina Bonciu, Florin Andrei, Alexandra Palla-Papavlu

**Affiliations:** National Institute for Lasers, Plasma and Radiation Physics, Atomistilor Street 409, 077125 Magurele, Romania

**Keywords:** laser transfer, LIFT, graphene oxide, PEDOT:PSS, hybrids, copper ions, voltammetry, screen-printed electrodes, sensor

## Abstract

Composites based on poly(3,4-ethylenedioxythiophene): poly(styrene sulfonate) (PEDOT:PSS)—graphene oxide (GO) are increasingly considered for sensing applications. In this work we aim at patterning and prototyping microscale geometries of PEDOT:PSS: GO composites for the modification of commercially available electrochemical sensors. Here, we demonstrate the laser-induced forward transfer of PEDOT:PSS: GO composites, a remarkably simple procedure that allows for the fast and clean transfer of materials with high resolution for a wide range of laser fluences (450–750 mJ/cm^2^). We show that it is possible to transfer PEDOT:PSS: GO composites at different ratios (i.e., 25:75 %wt and 50:50 %wt) onto flexible screen-printed electrodes. Furthermore, when testing the functionality of the PEDOT:PSS: GO modified electrodes via LIFT, we could see that both the PEDOT:PSS: GO ratio as well as the addition of an intermediate release layer in the LIFT process plays an important role in the electrochemical response. In particular, the ratio of the oxidation peak current to the reduction peak current is almost twice as high for the sensor with a 50:50 %et PEDOT:PSS: GO pixel. This direct transfer methodology provides a path forward for the prototyping and production of polymer: graphene oxide composite based devices.

## 1. Introduction

Quantifying biological or biochemical processes is of utmost importance for medical, biological, and biotechnological applications. However, converting biological information to an easily processed electronic signal is challenging due to the complexity of connecting an electronic device directly to a biological environment. Electrochemical sensors provide attractive means to analyze the content of a biological sample due to the direct conversion of a biological event to an electronic signal. Today, many sensors can be found in labs around the world, and a growing number are used as diagnostic tools in point-of-care testing [1].

Like many other micropollutants, heavy metals represent a growing environmental [2] and health [3] problem. Depending on the contamination pathway, they appear at detectable levels in food resources, thus contaminating humans [4]. Some heavy metals, such as copper (Cu), are known to play a vital role in physiological concentrations but can also be toxic in larger doses, i.e., Cu affects the liver and kidney [5]. In addition, copper ions are severely hazardous environmental pollutants with a toxic effect on living organisms due to their participation in producing reactive oxygen species [6]. According to the World Health Organization and European water quality standards, the concentration of copper in drinking water should not exceed 2 mg/L [7]. 

Heavy metal trace detection is mainly carried out using spectroscopic techniques: atomic absorption spectroscopy [8], inductively coupled plasma mass spectroscopy [9], X-ray fluorescence, quartz crystal microbalance [10], and neutron activation analysis are the most commonly used. However, they suffer from several significant drawbacks, including tedious sample preparation, the need for expert knowledge, high operational costs, and lengthy analysis time (calibration, pre-concentration, preparation, sampling) [11], which make them unsuitable for real-time online and continuous monitoring applications. On the contrary, biosensors [12] based on electrochemical devices represent an attractive alternative due to their numerous advantages. Electrochemical devices are mostly user-friendly since they require simple procedures, are low cost, and are well-suited for miniaturization and automatic in situ measurements with minimal sample changes. A significant challenge in heavy metal trace detection is the development of electrochemical techniques and devices that are robust, selective, have low detection limits, and allow fast analyses. The sensitivity and selectivity of the electrochemical sensing platform can be further improved by the chemical modification of bare electrodes with efficient electron mediators. It is essential that materials used as electrodes in electrochemical sensors have high surface reactivity and that their porosity can be modified so that the metal can be detected. A wide range of materials has already been used for electrode modification and copper determination. Titanium carbide (Ti_3_C_2_T*_x_*) and multiwalled carbon nanotubes nanocomposites [13], a three-dimensional l-cysteine self-assembled monolayer functionalized gold nanoparticles/single-walled carbon nanotubes/glassy carbon electrode [14], a 4-amino-6-hydroxy-2-mercaptopyrimidine monohydrate-based self-assembled monolayer [15], a polystyrene sulfonate–carbon nanopowders composite [16], a cysteine-functionalized graphene oxide (GO)/polypyrrole nanocomposite [17], graphene oxide sheets [18], tin oxide and palladium doped tin oxide (Pd:SnO_2_) [19], graphene oxide with L-cysteine [20], and a polypyrrole/reduced graphene oxide nanocomposite [21] have been used to make chemically modified electrodes to determine copper. 

The development of carbon-modified electrodes to increase the sensitivity limits of the electrochemical sensors has been gaining momentum [22]. In particular, smart materials [23], which include carbon materials organized into different structures such as carbon nanotubes, graphene, carbon dots, carbon cables, and nanodiamonds, have started to play a pivotal role in the nanotechnology field [24]. They are used in developing sensors, especially in composite designs, due to their high conductivity, high specific surface area, and excellent thermal stability. As a result of its high surface area, electron transfer capability, biocompatibility, and biomolecular affinity, graphene oxide makes the most attractive electrode material for the selective and sensitive detection of copper. It is noteworthy, however, that GO sheets contain abundances of oxygen-containing groups, such as epoxides, hydroxides, and carboxylic acids [25]. These functional groups allow the GO to disperse fairly well in an aqueous solution, but the electrical conductivity is reduced significantly due to the largely disrupted sp^2^ hybridized network [26]. Therefore, conductive polymers, such as poly(3,4-ethylenedioxythiophene) (PEDOT) were reported to enhance the electrical conductivity of GO [27]. One approach to strengthening electrochemical electrodes’ electrocatalytic activity is to integrate nanocomposites or hybrids, which can combine the best features of both components [28]. 

Incorporating carbon nanomaterials into PEDOT: PSS accelerates the stabilization of the polymer’s transport layers [29]. Furthermore, PEDOT:PSS might act as a suitable barrier for stopping the restacking process of GO layers, thus improving conductivity, electrocatalytic activity, and specific surface area [30]. It may therefore be possible to separate GO layers with a homogeneous dispersion of PEDOT: PSS and introduce the ability to use them as electrochemical sensor materials [31].

To detect Cu^2+^ and Pb^2+^, Kong’s lab developed a highly sensitive material based on graphene [32]. Gollavelli et al. demonstrated the feasibility of fabricating a magnetic graphene nanocomposite to remove heavy metal ions [33]. Nevertheless, high levels of other heavy metal ions can negatively influence the operation of these electrodes, such as those containing Pb(ii), Cd(ii), Ni(ii), and Co(ii). As a result of the van der Waals and π-π stacking interactions between adjacent graphene sheets, graphene becomes easy to agglomerate, reducing its advantage and limiting its use [34]. The quality of graphene used in electronic devices is highly critical, with very few defects and oxygenated groups. With cost-effective processing, graphene oxide (GO) sheets can be obtained directly from graphite after oxidation or exfoliation without undergoing further reduction. Given its large specific surface area and strong hydrophilicity, GO shows tremendous promise for removing aqueous pollutants, including heavy metal ions. In addition to serving as an excellent adsorbent, Cu^2+^ binds strongly to oxygen moieties on the GO surface, enhancing its electrical conductivity while also improving its chemical stability. 

The electrodes presented above have significant merits including high stability and reproducibility; however, their sometimes-inconvenient fabrication and high production costs may limit their application [35]. 

To date, few studies related to the use of PEDOT:PSS with graphene nanomaterials have been reported. The results obtained have shown that GO/PEDOT:PSS composites are promising candidates for modifying electrode material used in electrochemical sensing [36,37].

In this work we propose an original, flexible sensor based on a PEDOT: graphene oxide composite developed by a laser-based method, i.e., laser-induced forward transfer (LIFT), which has a low cost and can be successfully scaled. LIFT is a non-contact technique that may be used both for liquid-phase and solid-phase transfer [38,39]. The technique is suitable for applications where high spatial resolution is required, it doesn’t require complex masks, as in lithography, and where it is not limited by the rheological properties of the material under transfer, providing the ability to print materials within a broad range of viscosities ranging from low viscosity Newtonian fluids (water) to non-Newtonian fluids (pastes). It involves two substrates, i.e., the donor substrate coated with the material of interest and a receiver substrate, which are brought in close proximity (<10 µm). During LIFT, a laser beam is imaged at the interface between the transparent laser support and the material to transfer (also named donor). Generally, the mechanism for solid-phase printing involves a single laser pulse that irradiates the donor substrate, and the thermally induced stresses introduced on the donor surface lead to the ejection of the solid flyer mimicking the shape of the laser spot [40]. Recently, LIFT has been applied for printing Au voxels as porous electrodes. The authors reported that the LIFT-ed electrodes presented an increase in the electrochemically active surface area by a factor of four compared with a sputtered dense Au film when characterized using cyclic voltammetry in Ar-saturated 0.1 M KOH [41]. In addition, in [42], a screen-printed electrode modified by LIFT of different liquid mixtures was demonstrated for the detection of organophosphorous and carbamate pesticides. 

Moreover, in order to improve the process efficiency and to protect the material to be transferred, a sacrificial layer, called dynamic release layer (DRL), which is a metal or polymer film of tens of nanometers in thickness, is introduced between the donor substrate and the material of interest to absorb the laser energy and induce the transfer process. 

In this work, we carry out transfers with and without a triazene polymer (TP) layer as a DRL [43]. Triazene is highly photoactive and strongly absorbs in the UV spectrum with a maximum absorbance of around 200 nm, making it a good candidate for a release layer. In this work, the TP layer is placed between the quartz substrate and the PEDOT:PSS: GO thin layer. Upon laser interaction, the TP layer decomposes, allowing the transfer of the PEDOT:PSS: GO layer onto a substrate (i.e., flexible screen printed electrodes) placed parallel and in close proximity to the donor. Our work seeks to demonstrate the applicability of laser-based methods for the realization of proof of concept cost effective sensors based on polymer: graphene oxide composites for copper ion monitoring, thus paving the way for innovative and affordable cooper ions monitoring routes in complex biomatrices such as blood, urine, or saliva.

## 2. Materials and Methods

### 2.1. Preparation of Donor Films

The materials used in this study for the preparation of the donor films are Graphene Oxide (GO) dispersed in isopropyl alcohol (1:1) at a 1 mg/mL concentration, supplied by The Graphene Box, Spain, poly(3,4-ethylenedioxythiophene): poly(styrene sulfonate) (PEDOT:PSS) obtained from Heraeus (Clevios PVP AI4083) with a PEDOT:PSS concentration of 1.3% *w*/*w* (weight ratio of PSS to PEDOT = 6)), and the triazene polymer, synthesized as described in [43]. All the chemicals used for the preparation of the donor films are of analytical grade and are used with no further purification. 

The PEDOT:PSS: GO nanocomposite blends are prepared by adding the GO dispersion to the PEDOT:PSS solution, magnetically stirring the blend for 90 min and sonicating it for 15 min at room temperature. Two concentrations are prepared, i.e., 25% wt/wt PEDOT:PSS: GO and 50% wt/wt PEDOT:PSS: GO.

Two types of donor films are prepared, i.e., single-layer PEDOT:PSS: GO films and multilayer TP: PEDOT:PSS: GO films. Both types of donor films are prepared by spin-coating. 

The single-layer donor films consist of an excess amount of PEDOT:PSS: GO solution applied to the 2.5 mm × 2.5 mm quartz substrate (manual using a syringe and distributed in the cover of the coating device by centrifugation). The quartz substrate is then rotated at 500 rpm for 30 s, followed by 1500 rpm for 30 s to disperse the fluid by centrifugal force. The rotation is continued as the fluid rotates to the edges of the substrate. 

The multilayer donor films are prepared in two steps. First, a triazene polymer solution (i.e., TP in chlorobenzene/cyclohexanone (1:1, *w*/*w*)) is spin coated onto the quart substrate at 2000 rpm/min, resulting in films of 100 nm thickness. Second, the PEDOT:PSS: GO solution is spread onto the quartz substrate coated with a TP film and spin coated at 1500 rpm for 30 s.

Both the single and multilayer PEDOT:PSS: GO donor films are placed on a hot plate (50 °C/30 min) to initially evaporate the solvent and solidify the coating.

### 2.2. Laser-Induced Forward Transfer (LIFT)

The LIFT setup used in this work consists of a pulsed ArF laser (193 nm emission wavelength, 15 ns pulse length, 1 Hz repetition rate), which is guided and imaged with an optical system onto the donor substrate to transfer PEDOT:PSS: GO micro pixels from the donor substrate to the receiving surface. A computer-controlled XYZ translation stage allows the displacement of both donor and receptor substrates with respect to the laser beam, the donor, and the receiver being placed in close contact (<1 µm). A laser pulse incident on the back side of the transparent carrier is imaged by a lens onto the donor material, yielding a spot size of 1.2 mm in diameter, and by means of shock formation and ablation, propels a small part of the donor film forward, resulting in the deposition of the PEDOT:PSS: GO pixels onto the substrate. The laser fluence is varied between 450 mJ/cm^2^ and 750 mJ/cm^2^. All experiments are carried out under ambient pressure at temperatures close to room temperature. 

The receiver substrates are flexible, disposable, and low-cost screen-printed (SPE) electrodes from Dropsens (ref. ITO10, Spain) with a carbon counter electrode (CE), a reference electrode (RE) made of silver, and a working electrode (WE) based on ITO. The WE consisting of 3 mm diameter ITO disk of identical commercial electrodes is modified with different PEDOT:PSS: GO pixels using a laser-induced forward transfer.

### 2.3. Characterization of the Laser-Transferred Materials and the As-Deposited Sensors

Topographical investigations of the transferred pixels as well as the donor films prior to ablation are carried out by means of scanning electron microscopy (SEM) with a JSM-531 Inspect S. system (Hillsboro, OR, USA) at a voltage of 20 kV. For the SEM analysis, the layers are covered using a sputter coater with 10 nm Au (Agar Scientific Ltd., Essex, UK).

The layers’ surface morphology and overall roughness (quantified by the root mean square roughness) are analyzed by atomic force microscopy (AFM) (XE 100, Park Systems, Suwon, South Korea). Imaging is realized in non-contact mode, using silicon tips, in ambient conditions.

The wettability of the PEDOT:PSS: GO pixels transferred onto the commercial electrodes is evaluated by contact angle measurements with a KSV CAM101 microscope equipped with a video camera. All contact-angle measurements are obtained by dispersing double-distilled water droplets with a volume of 2.5 ± 0.5 μL. Five different points are measured for every thin film, and the contact angle reported is the average of these measurements.

The electrochemical investigations are realized with an AutoLab PGSTAT302N controlled by NOVA 1.11 software. The commercial screen-printed electrode consists of an ITO working and auxiliary electrode and a silver reference electrode. In the electrode modification procedure, only the working electrode is coated by LIFT with a PEDOT:PSS: GO pixel. The connection between the potentiostat and the electrodes is made using a standard cable connector for screen-printed electrodes. All the sensors are tested for copper detection by cyclic voltammetry measurements scanning the potential from −1.4 V to 0.4 V and reversely using a scan rate of 100 mV/s. An amount of 100 ppm of Cu^2+^ is added to 0.1 M acetate buffer solution (pH = 5.0) and is energetically stirred for 20 min. The amount of copper is electrochemically detected by placing 50 μL of solution over the electrode surface. The same procedure is used for testing all of the sensors. All of the tests were carried out at room temperature. 

## 3. Results and Discussion

### 3.1. Donor Film Fabrication

**Figure 1:**
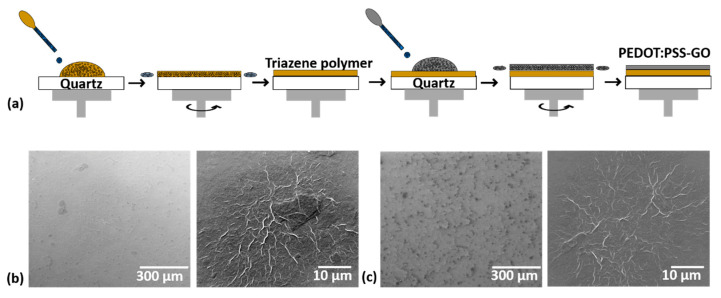
(**a**) Schematic representation of the experimental multilayer donor fabrication steps. In the case of single donor layer fabrication, the triazene polymer spin coating step is missing. Top-view SEM micrographs of PEDOT:PSS: GO layers with different concentrations (**b**) 25:75 wt.% and (**c**) 50:50 wt.% deposited by spin-coating on quartz.

The first step taken to modify electrochemical electrodes with a PEDOT:PSS: GO blend via laser-induced forward transfer is to obtain uniform and homogeneous donor films. Here, this is achieved by the spin-coating process (a scheme of the procedure is shown in Figure 1a). We carry out studies on the influence of substrate rotation speed and rotation time on the thickness and morphology of the resulting donor films. The experimental spin coating sequence chosen is 500 rpm for 30 s, followed by 1500 rpm for 30 s. The thickness of the single layer/multilayer donor films determined by at least 5 AFM measurements along scratch lines on each sample is 80 nm in the case of the PEDOT:PSS: GO coating and 180 nm for the TP/PEDOT:PSS: GO coating. Understanding the role of GO in the PEDOT:PSS matrix is very important to simplify the spin coating process and to improve the performance of the electrodes. Thus, we carried out a detailed investigation of the surface morphology of the as-deposited donor films. 

The micro and nano organization/morphology of all deposited coatings is evaluated by SEM. Complete and uniform coating of the substrate surface is obtained by centrifugation, with the surface morphology of the coatings depending on the change of the dispersion’s composition (Figure 1b,c). Not only the uniformity of the coatings obtained by spin coating is observed, but also the uniform distribution of the graphene oxide sheets in the composite layers with the increasing proportion of GO. We could obtain a good dispersion of the GO into PEDOT:PSS, and both concentrations, i.e., 25:75 wt.% PEDOT:PSS: GO and 50:50 wt.% PEDOT:PSS: GO lead to continuous films with some GO aggregates visible, and by increasing the GO content up to 50% wt. the presence of the aggregate increases, which can be seen in Figure 1b,c.

### 3.2. Laser Printing of PEDOT:PSS: GO Composites

The objective of this study is to demonstrate the fabrication of a proof-of-concept electrochemical sensor based on polymer: graphene oxide composites for copper ion monitoring. Here we focus on investigating the surface morphology of the transferred composite under different laser fluences as well as when applying an intermediate sacrificial layer as compared to the case of a direct transfer (without an intermediate layer). 

**Figure 2:**
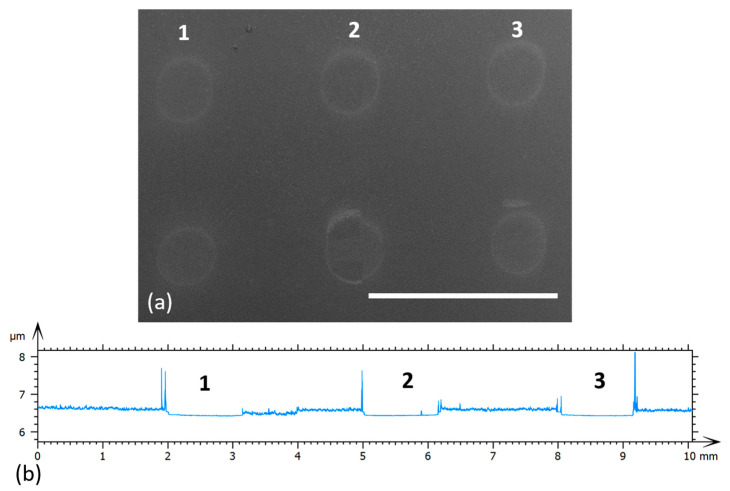
(**a**) Top-view SEM micrographs of 25:75 wt.% PEDOT:PSS: GO pixels transferred at different laser fluences, i.e., from left to right, 450 mJ/cm^2^, 600 mJ/cm^2^ (middle), and 750 mJ/cm^2^ on glass substrates. The transfer is carried out with the donor and receiver substrates in contact. The scale bar is 3 mm. (**b**) Profilometry traces of the ablated spots created by a single pulse in the 80 nm thick donor film of PEDOT:PSS: GO at three increasing laser fluences of 450, 600, and 750 mJ/cm^2^ (from right to left). The profilometry traces correspond to pixels 1, 2, and 3 from (**a**).

First, the influence of laser fluence on the PEDOT:PSS: GO transferred material is discussed, in order to obtain a regular, debris free transfer. The constant layer thickness of 80 nm PEDOT:PSS: GO w/wo a 100 nm triazene polymer layer are selected to study the laser fluence influence on the transferred pixels. Top view SEM images of 25:75 wt.% PEDOT:PSS: GO pixels transferred between 450 and 750 mJ/cm^2^ on a glass substrate are shown in Figure 2a. The transfers are carried out with the donor and receiver substrates in contact. As it can be seen from Figure 2a, transferring PEDOT:PSS: GO pixels is possible, leading to transferred material visible on the receiver substrate, the best transfers being achieved with a laser fluence range of 450–750 mJ/cm^2^. The transfer fluence above 750 mJ/cm^2^ leads to splashes around the transferred material, while no transfer can be noticed below 450 mJ/cm^2^. 

Further on, we investigate the possibility of transferring PEDOT:PSS: GO composites with a TP intermediate layer. Soft composites such as PEDOT:PSS: GO may be easily damaged by the laser pulses, resulting in a loss of functionality. Therefore, in order to reduce the risk of damaging the layer or the surface of the layer to be transferred and to improve the process efficiency, the donor substrate is precoated with a sacrificial triazene polymer as intermediate layer (placed between the quartz substrate and the PEDOT:PSS: GO layer). From the scanning electron microscopy observations, transferring PEDOT:PSS: GO with the help of a TP DRL led to well defined pixels with a flat appearance, without fragmented deposits at laser fluences in the range 450–750 mJ/cm^2^, similar to the case of PEDOT:PSS: GO transfers without a TP intermediate layer (not shown here). In addition, the profilometry traces of the ablated spots created by a single pulse in the 180 nm thick donor film of TP/PEDOT:PSS: GO at 3 increasing laser fluences of 450, 600, and 750 mJ/cm^2^ (from right to left) is shown in Figure 2b.

**Figure 3:**
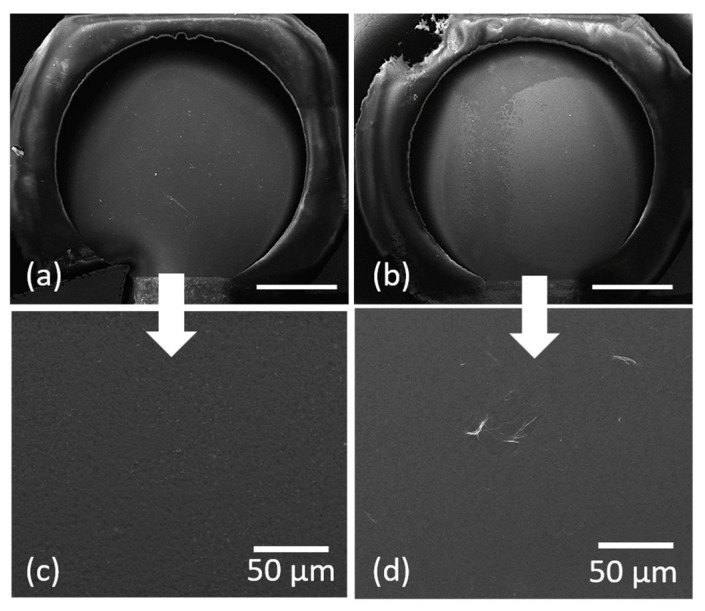
SEM images taken at different magnifications of the top surface of the working electrode (**a**,**c**) prior to LIFT; (**b**,**d**) after printing a 25:75 wt.% PEDOT:PSS: GO pixel at 600 mJ/cm^2^ with a triazene polymer intermediate layer. The transfers are carried out at room temperature and in close contact between the donor and the receiver. The scale bar in **a**,**b** is 1 mm.

The next step to fabricate the electrochemical sensor is to transfer the polymer: graphene oxide composites onto the commercial screen-printed electrodes. An example of a PEDOT:PSS: GO pixel transferred with a TP intermediate layer is shown in Figure 3. Each working electrode is printed with 2 PEDOT:PSS: GO pixels, placed one next to the other (thus covering approximately 32% of the surface of the working electrode). 

**Figure 4:**
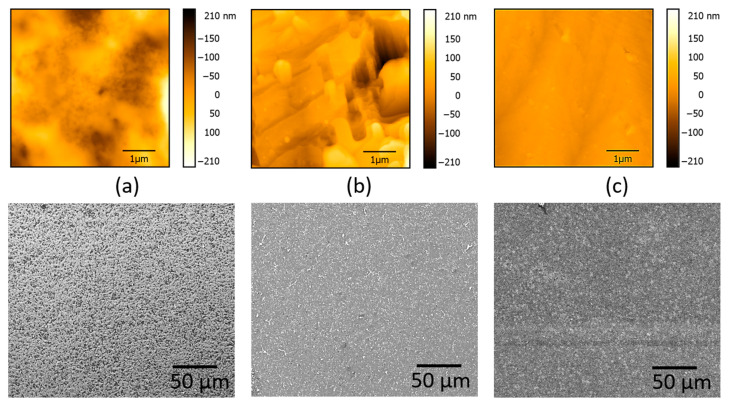
(**a**) AFM and SEM images of the top surface of the electrode prior to LIFT; (**b**) AFM and SEM images of the top surface of the electrode after printing a 25:75 wt.% PEDOT:PSS: GO pixel at 600 mJ/cm^2^ without an intermediate triazene polymer layer; the root-mean-square roughness (RMS) value determined from the AFM is 45 nm; (**c**) AFM and SEM images of the top surface of the electrode after printing a 50:50 wt.% PEDOT:PSS: GO pixel at 600 mJ/cm^2^ without an intermediate triazene polymer layer; the RMS value is 11 nm. The transfers are carried out at room temperature and in close contact between the donor and the receiver.

Furthermore, AFM and SEM images of the top surface of the electrodes after printing 25:75 wt.% and 50:50 wt.% PEDOT:PSS: GO pixels at 600 mJ/cm^2^ are shown in Figure 4a–c. The difference in surface topography when increasing the GO concentration is attributed to the increased wrinkling of the GO flakes, as can be seen in Figure 4. During the donor fabrication steps, both vacancies and topological defects are introduced in the carbon plane of the GO, leading to a richer texture in terms of rippling and wrinkling. The generation of these wrinkles may be attributed to the epoxy groups found on the basal plane of GO, which have the tendency to form chains on the surface of GO leading to the formation of topological defects and distortions that line up along the wrinkles path [44]. The SEM images show that the transferred hybrid films using 600 mJ/cm^2^ are clean and have a relatively uniform morphology without any apparent defects. This indicates that there is no sign of agglomeration of GO in composite samples and that most of the graphite sheets are dispersed homogeneously into the PEDOT:PSS polymer matrix. Therefore, the morphology of the transferred pixels suggests that the interfacial interaction between PEDOT:PSS and GO is rather strong with a very low degree of agglomeration of the GO sheets. 

**Figure 5:**
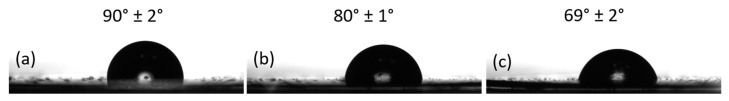
Contact angle images for (**a**) the uncoated working electrode, (**b**) the working electrode coated with a 25:75 wt.% PEDOT:PSS: GO pixel and (**c**) the working electrode coated with a 50:50 wt.% PEDOT:PSS: GO pixel transferred at 600 mJ/cm^2^.

Contact-angle measurements are widely used for the characterization of the wettability of PEDOT:PSS: GO on the SPE electrodes (Figure 5). The uncoated SPE electrode has the highest contact angle, i.e., 90 ± 2°. After transferring the PEDOT:PSS: GO blend onto the SPE electrodes, the contact angles decrease. For the 50:50 wt.% PEDOT:PSS: GO films, a minimum contact angle of approx. 70° is observed, which ensures a good coverage of the electrodes. This could be attributed to the fact that the water soluble PSS part is a good dispersant, thus allowing the improvement of the dispersion of GO in the PEDOT:PSS polymer matrix [45,46].

### 3.3. Electrode Modification via LIFT—Influence of Laser Fluence

**Figure 6:**
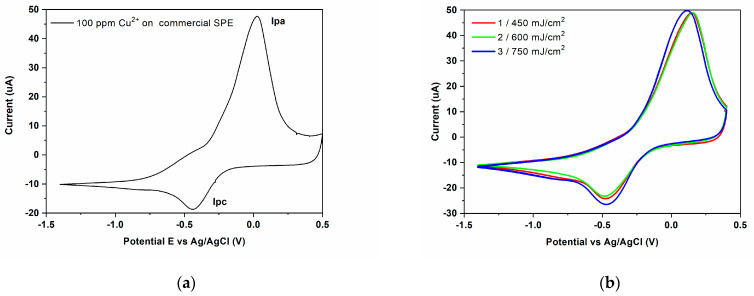
(**a**) Cyclic voltammogram of Cu^2+^ (100 ppm) in 0.1 M acetate buffer solution (pH = 5.0) recorded on the commercial SPE based PEDOT (**b**) Cyclic voltammogram of Cu^2+^ (100 ppm) in 0.1 M acetate buffer solution (pH = 5.0) recorded on PEDOT:PSS: GO sensors fabricated at different laser fluences: 1 = 450 mJ/cm^2^; 2 = 600 mJ/cm^2^; 3 = 750 mJ/cm^2^.

Given that the scope of the paper is the demonstration of proof-of-concept electrochemical sensors for the detection of copper ions, we carried out functionality tests of the LIFT-fabricated PEDOT:PSS: GO working electrodes. The functionality tests are carried out by evaluating the response of the laser printed electrodes in different experimental conditions at 100 ppm Cu^2+^. In particular, the experimental conditions investigated are the influence of the laser fluence, the polymer: graphene oxide ratio, and the addition of an intermediate triazene polymer layer for printing the polymer: graphene oxide composite. The cyclic voltammogram obtained for the utilization of bare (untreated) flexible SPE working electrode for the electrochemical detection of 100 ppm Cu^2+^ in 0.1 M acetate buffer solution is presented in Figure 6a. During the forward scan, at around −430 mV, a single cathodic peak (I_pc_), correlated to the Cu^2+^ reduction via two-electron transfer reaction (Cu^2+^ + 2e^−^ → Cu^0^) is obtained. Taking into account that the reaction medium has a pH = 5, the bivalent copper is directly reduced to metallic copper [47]. For the reverse scan, the oxidation of metallic copper through a one-step oxidation reaction is observed with an anodic peak (I_pa_) at +26 mV. For all untreated and modified electrodes, data from current vs. potential plots are gathered in Table 1.

When studying the influence of the laser fluence on the printed PEDOT:PSS: GO blend, it has been found that the electrochemical properties of PEDOT:PSS: GO sensors fabricated at different laser fluences are quite similar, the positions of anodic and cathodic peak potentials being slightly shifted between 110–150 mV and 470–480 mV, respectively (Figure 6b). For samples 1 and 2, the values of I_pa_ are smaller, while for sample 3 the value of I_pa_ is similar compared to the commercial electrode, while I_pc_ values increase up to 63% (for sample 3, i.e., the PEDOT:PSS: GO pixel transferred at 450 mJ/cm^2^) as compared to the bare sensor. 

The current ratio i_pa_/i_pc_ is another important parameter which can be extracted from cyclic voltammograms. It gives information about the chemical stability of the electrochemically generated product and its stability is demonstrated by a peak current ratio of unity. It also gives information about the reversibility of the system. The reversibility of a redox system is correlated to the “rapidity” of the analyte to exchange electrons at the electrode. For completely Nernstian systems (reversible redox systems), the electron transfer occurs quickly without significant thermodynamic barriers (electron transfer rate ≫ mass transfer rate of diffusion). In addition, the theoretical value for the separation between the two peak potentials, ΔE_p_ is equal to 59/n mV, regardless of scan rate, for a reversible process. Moreover, the ratio of peak currents for reversible systems is equal to unity (i_pa_/i_pc_ = 1) [47,48]. 

The increase of the i_pa_/i_pc_ ratio can be correlated to a lower reversibility of the system. All fabricated working electrodes through the LIFT technique show higher reversibility than the commercial electrode, except for the sensor prepared with 50:50 wt.% PEDOT:PSS: GO, without TP, at a laser fluence of 600 mJ/cm^2^, which shows a i_pa_/i_pc_ = 5.01. However, in our case, for all tested sensors, both oxidation and reduction peaks are obtained. The peak potential separation (∆E_p_) is between 580–630 mV and the ratio of the oxidation peak current to the reduction peak current is about 1.97–2.79, values similar to those reported by Shaikh et. al. [49], but higher compared to those reported by Haque et. al. [50]. The values of the separation between the two peak potentials, ΔE_p_ is different than 59/n mV and i_pa_/i_pc_ > 1, so we can assume that the redox system of copper at the graphene composite is a quasi-reversible one.

### 3.4. Electrode Modification via LIFT—Influence of Polymer: Graphene Oxide Ratio

**Figure 7:**
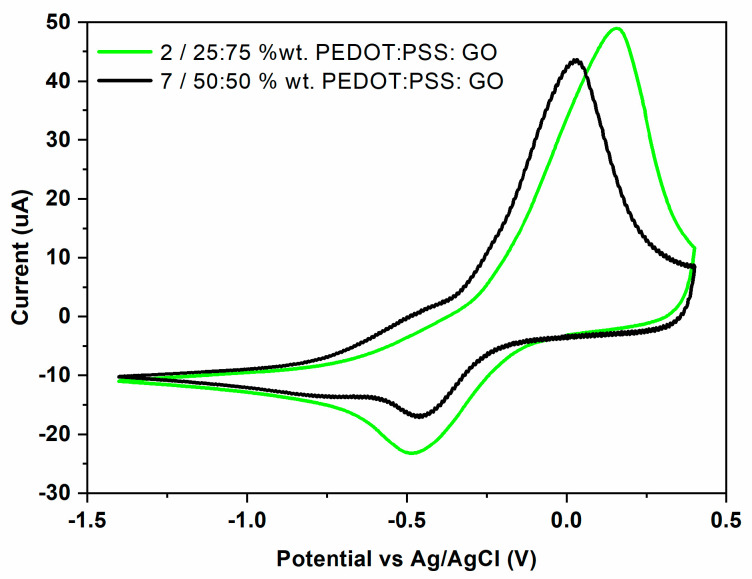
Cyclic voltammogram of Cu^2+^ (100 ppm) in 0.1 M acetate buffer solution (pH = 5.0 upH) recorded on PEDOT:PSS: GO sensors with different polymer: graphene oxide ratios, i.e., graph 2 = 25:75 wt.% PEDOT:PSS: GO, and graph 7 = 50:50 wt.% PEDOT:PSS: GO.

The polymer: graphene oxide ratio in the transferred pixel plays an important role on the electrochemical response of the PEDOT:PSS: GO modified SPE sensors (Figure 7). For example, when a 25:75 wt.% PEDOT:PSS: GO (sample 2) ratio is utilized for sensors fabrication, the resulting device shows an intense oxidation peak with a value of I_pa_ = 40.66 μA and I_pc_ = 14.59 μA. By increasing the polymer concentration to a ratio of 50:50 wt.% PEDOT:PSS: GO, a decrease of both peak currents is observed. At the same time, the anodic peak potential is shifted to more negative potentials, while the cathodic peak potential is shifted to positive potentials. As can be observed in Table 1, the peak potential separation decreases with polymer concentration, while the ratio of the oxidation peak current to the reduction peak current is almost twice as high for the sensor that is richer in the polymer.

**Figure 8:**
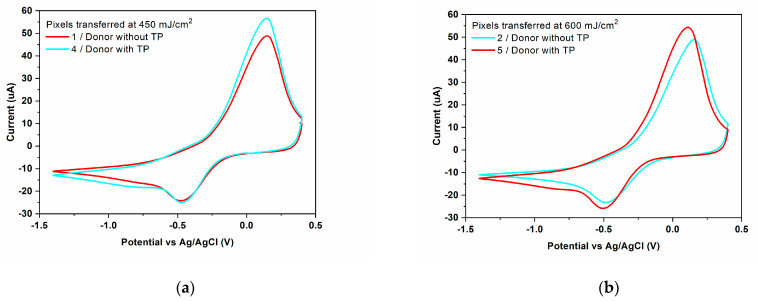
Cyclic voltammogram of Cu^2+^ (100 ppm) in 0.1 M acetate buffer solution (pH = 5.0 upH) recorded on PEDOT:PSS: GO electrodes printed from a donor with a triazene polymer intermediate layer and fabricated at different laser fluences (**a**) laser fluence 450 mJ/cm^2^; (**b**) 600 mJ/cm^2^.

The addition of an intermediate triazene polymer layer in the laser transfer process has a major impact on the electrochemical behavior of the final device. As can be observed in Figure 8, for the sample printed with triazene, the anodic current increases up to 48.84 μA (the PEDOT:PSS: GO electrode printed at a laser fluence of 600 mJ/cm^2^). The same sample shows a slight increase of the I_pc_ compared to the value recorded by the sensor printed without triazene and a decrease of the oxidation peak current to the reduction peak current. In addition, we have found that the values of I_pa_ and I_pc_ obtained for the sensors printed with a triazene polymer intermediate layer are higher compared to the SnO_2_ and Pd-SnO_2_ based sensors reported in [19]. These results are in good agreement with results reported in the literature [42], which also show that the addition of a triazene polymer layer is an important parameter for obtaining a regular, “clean” transfer. Moreover, the triazene polymer, as an intermediate layer, absorbs the laser radiation and subsequently decomposes into gaseous fragments which are used to transform the energy into a required mechanical push. Thus, we could assume that the presence of the TP layer in the donor improves the interfacial adhesion of the pixel to the SPE electrode, which is in turn a basic factor to obtain a good working electrode in electrochemical sensor applications. 

**Figure 9:**
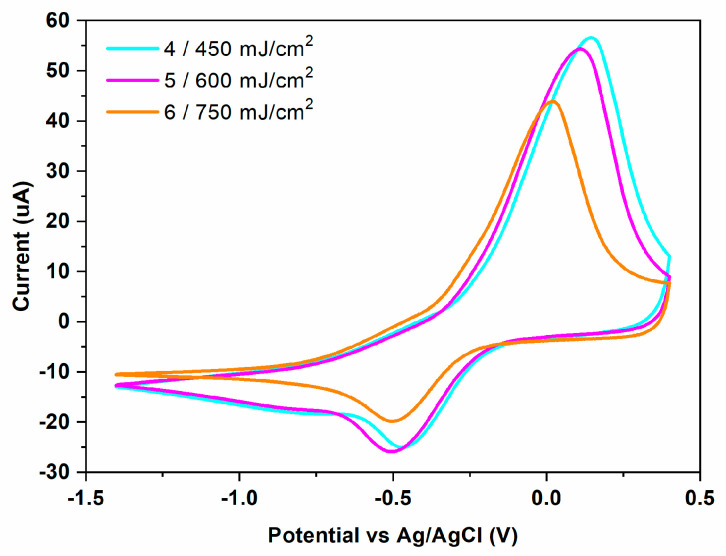
Cyclic voltammogram of Cu^2+^ (100 ppm) in 0.1 M acetate buffer solution (pH = 5.0 upH) recorded on PEDOT:PSS: GO modified SPE electrodes fabricated at different laser fluences via LIFT from a donor film with an intermediate triazene polymer layer.

An interesting trend is observed for triazene based sensors fabricated at different laser fluences (Figure 9). Both I_pa_ and I_pc_ decreases with the increase of the laser fluence, and peak potentials are shifted toward negative potentials (for E_pa_) and positive potentials (for E_pc_), respectively.

It can be concluded that the functionality tests carried out with the LIFT modified commercial SPE electrodes, i.e., with a PEDOT:PSS: GO blend are feasible for the detection of copper ions. 

## 4. Conclusions

In this work, a laser-induced forward transfer (LIFT) method was optimized for printing polymer: graphene oxide (GO) pixels aiming at the fabrication of electrochemical sensors for the detection of copper ions. The pixels were produced by transferring poly(3,4-ethylenedioxythiophene): poly(styrene sulfonate) (PEDOT:PSS): GO composite on the working electrode of a commercial electrochemical flexible sensor. Our approach is simple and flexible due to the fact that PEDOT:PSS:GO composites at different ratios can be easily deposited by LIFT on various supports, including flat and non-conformal plastics, and there is no need for prior surface functionalization.

Surface characterization of the transferred features using atomic force microscopy (AFM) and scanning electron microscopy (SEM) corroborated that the PEDOT:PSS:GO composite was transferred in a “clean” and regular manner, with a high resolution on the surface of the working electrode of a commercial electrochemical flexible sensor. Furthermore, by applying the PEDOT:PSS: GO composite on the working electrode, a decrease of the contact angle can be measured, i.e., from 90° to 70° for the SPE printed with a 50:50 wt.% PEDOT:PSS: GO pixel.

We were able to show for the first time the transfer of polymer: graphene oxide composites as thin-pixels onto flexible, working electrodes and prove their functionality by testing them against copper ions. As copper ion detection has received great attention in recent years, we intend to expand our studies and investigate the performance of our modified electrodes towards different concentrations of copper ions further. In addition, it will be interesting to determine the minimum quantity of composite on the surface of the commercial electrode which renders the highest effect towards copper ion determination. We believe that this study, i.e., the demonstration of modifying working electrodes via laser-based methods for the determination of copper ions, might represent a solution for potential applications in diagnostic tools for point of care testing.

## Figures and Tables

**Table 1:** Data gathered from the current vs potential plots obtained for the commercial working electrode and the LIFT fabricated working electrodes.

Sensor	Experimental Conditions	Peak Current (μA)		Peak Potential (mV)		∆E_p_ = E_pa_ − E_pc_	i_pa_/i_pc_
		(−) i_pc_	i_pa_	(−) E_pc_	E_pa_		
Commercial sensor		11.41	42.9	430	26	450	3.76
1 *	25:75 wt.% PEDOT:PSS: GO Donor without TP 450 mJ/cm^2^	17.09	35.32	470	140	610	1.97
2 *	25:75 wt.% PEDOT:PSS: GO Donor without TP 600 mJ/cm^2^	14.59	40.66	480	150	630	2.79
3 *	25:75 wt.% PEDOT:PSS: GO Donor without TP 750 mJ/cm^2^	17. 9	42.98	470	110	580	2.51
4 *	25:75 wt.% PEDOT:PSS: GO Donor with TP 450 mJ/cm^2^	17.34	47.94	460	150	610	2.76
5 *	25:75 wt.% PEDOT:PSS: GO Donor with TP 600 mJ/cm^2^	16.14	48.84	500	110	610	3.02
6 *	25:75 wt.% PEDOT:PSS: GO Donor with TP 750 mJ/cm^2^	11.84	39.52	500	10	510	3.33
7 *	50:50 wt.% PEDOT:PSS: GO Donor without TP 600 mJ/cm^2^	7.72	38.68	460	22	480	5.01
SnO_2_	Ref. [19]	10.01	38.03	460	−31	429	2.90
Pd-SnO_2_	Ref. [19]	15.18	44.06	340	110	450	3.79

* this work.

## Data Availability

The data used to support the findings of this study are available from the corresponding author upon request.
